# Atezolizumab for the First-Line Treatment of Non-small Cell Lung Cancer (NSCLC): Current Status and Future Prospects

**DOI:** 10.3389/fonc.2018.00277

**Published:** 2018-07-24

**Authors:** Rachel Ryu, Kristina E. Ward

**Affiliations:** Department of Pharmacy Practice, College of Pharmacy, University of Rhode Island, Kingston, RI, United States

**Keywords:** immunotherapy, targeted therapy, non-small cell lung cancer, squamous cell cancer, large cell carcinoma, adenocarcinoma

## Abstract

**Purpose:** Atezolizumab is a programmed death ligand 1 (PDL-1) blocking antibody that was approved for metastatic non-small cell lung cancer (NSCLC) in patients with disease progression. Various studies have been initiated to explore the effectiveness of atezolizumab among different patient cohorts and disease statuses, including as first-line therapy. The purpose of this paper is to identify and summarize the trials that use atezolizumab as a first-line agent in chemotherapy-naïve patients with NSCLC.

**Methods:** A database search was performed on Pubmed, Embase, and Wiley Cochrane Library—Central Register of Controlled Trials to identify clinical trials using atezolizumab as first-line therapy in NSCLC. Additionally, ClinicalTrials.gov and the International Clinical Trials Registry Platform (ICTRP) were searched to identify relevant clinical trials. Conference abstracts from the American Society of Clinical Oncology, the European Society for Medical Oncology, and the American Association for Cancer Research were hand-searched. Any trial in which atezolizumab was used as first-line therapy in chemotherapy-naive patients with NSCLC was included.

**Results:** Fifteen studies were ultimately included, all of which are current and ongoing. Of the 15 studies, 5 have reported results. When given in the first-line setting, atezolizumab had higher rates of objective response, progression-free survival, and overall survival, compared to the second and third-line settings. Among the 15 studies, atezolizumab is used as monotherapy (*n* = 5), in combination with chemotherapy (*n* = 6), in combination with targeted therapy such as bevacizumab (*n* = 1), as neoadjuvant/adjuvant therapy (*n* = 3), in combination with stereotactic body radiation therapy (*n* = 1), and in combination with or following chemoradiation (*n* = 1).

**Conclusion:** Available evidence shows promising safety and efficacy with the use of atezolizumab as first-line therapy in NSCLC. Atezolizumab is currently being studied in a variety of treatment settings. If clinical benefits are shown, atezolizumab may deem to be a useful first-line agent in NSCLC.

## Introduction

The levels of activity, response, and differentiation of T cells are largely regulated by positive and negative effects on the costimulatory pathways (1). Activation of T cells occurs when the co-stimulatory molecules, B7-1 and B7-2, bind to the CD28 receptor (2). T cells are inhibited when the B7-1 and B7-2 molecules bind to the cytotoxic T cell lymphocyte antigen 4 (CTLA-4). Inhibition also occurs when other ligands of the same B7 family, B7H1 (PDL-1) and B7-DC (PDL-2), bind to the PD-1 receptor (1, 2). B7H-1 is predominately found in tumors cells and rarely in human tissue (3). Therefore, when tumor-associated B7H-1 molecules bind to PD-1 receptors on activated T cells, T cells undergo apoptosis, thereby disabling the immune system from actively killing tumor cells (3, 4).

Atezolizumab is a PDL-1 inhibitor that was designed to restore anti-tumor action by preventing the immunosuppression caused by the B7H-1 and the PD-1 interaction (5). Atezolizumab was approved by the Food and Drug Administration (FDA) in October 2016 for the treatment of metastatic non-small cell lung cancer (NSCLC) in patients whose disease progressed during or following traditional platinum-based therapy, or targeted therapy in patients harboring the epidermal growth factor receptor (EGFR) mutation or anaplastic lymphoma kinase (ALK) fusion. Approval was based on the findings of the POPLAR and OAK trials, which administered atezolizumab for the second-line treatment of advanced NSCLC, and longer rates of overall survival were shown (6, 7).

The status of atezolizumab in comparison to other immune checkpoint inhibitors employing the PDL-1 signaling pathway is worth noting. Pembrolizumab is approved as first-line therapy for patients with NSCLC expressing over 50% PDL-1 expression, whereas nivolumab is limited for use in patients with metastatic NSCLC or disease progression. Durvalumab is indicated for use in patients with unresectable stage III NSCLC following chemoradiation with no signs of disease progression. Lastly, avelumab has not yet been approved for use in NSCLC, though research is ongoing. In a phase 1b trial of 145 patients with advanced NSCLC without the EGFR mutation or ALK fusion and not preselected for PDL-1 expression, improved response rates were seen with avelumab as first-line therapy (8). The results of a phase III trial comparing first-line avelumab to standard chemotherapy in patients with NSCLC is pending. The objective of this paper is to identify and summarize all trials that use atezolizumab as a first-line agent in chemotherapy-naïve patients with NSCLC.

## Materials and methods

This paper was conducted in accordance with the Preferred Reporting Items for Systematic Reviews and Meta-Analyses (PRISMA) guidelines and was prospectively registered with PROSPERO (registration number: CRD42017069416). A database search was performed on Pubmed (1946–2017), Embase (1947–2017), and Wiley Cochrane Library—Central Register of Controlled Trials (1898–2017) to search for clinical trials using atezolizumab as first-line therapy in NSCLC. Search strategies included controlled vocabulary and terms such as “nonsmall cell lung cancer,” “squamous cell cancer,” “large cell carcinoma,” and “adenocarcinoma.” Limits on year were not placed and studies were limited to English. Additionally, ClinicalTrials.gov and the International Clinical Trials Registry Platform (ICTRP) were searched to identify relevant clinical trials. Databases and clinical trial registries were last searched on 5/17/17. An example of the search strategy for Pubmed is shown in Appendix 1. Journals such as the American Society of Clinical Oncology (2004—present), the European Society for Medical Oncology (2004—present), and the American Association for Cancer Research (2004—present) were last accessed on 5/22/18.

## Results

**Figure 1:**
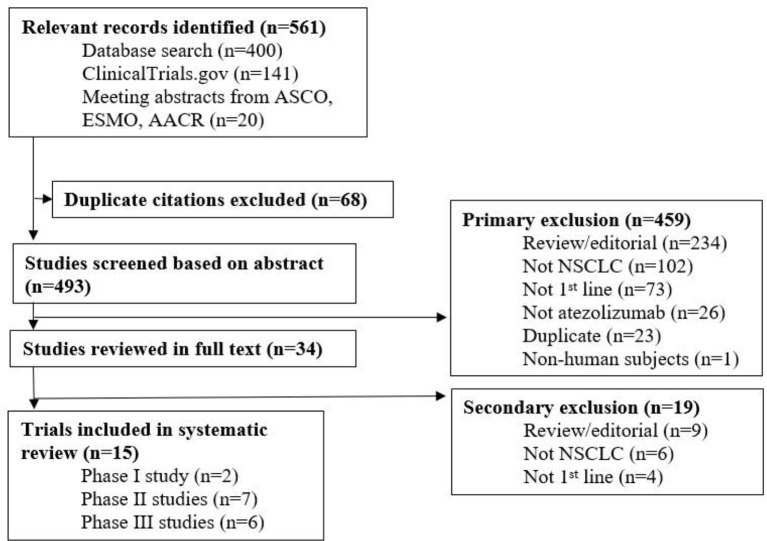
Screening and eligibility evaluation phases.

A total of 561 records were identified from all sources and duplicates were subsequently removed. The remaining studies were screened for relevancy and of those, 459 studies were excluded. Thirty-four studies were reviewed in full text and 15 studies were ultimately included (Figure 1). Among the 15 ongoing studies, 5 have reported results (Table 1).

**Table 1:** Current trials with results.

**Identifier, phase**	**Disease**	**Intervention**	**Results**			
			**Efficacy analysis**	**ORR**	**PFS (months)**	**OS (months)**
NCT01633970^b^ 1b	IIIB, IV, or recurrent NSCLC	Arms A, B, F^*^ **Arm C: Atez** + **PC Arm D: Atez** + **Pem-Cb Arm E: Atez** + **nab-PC**	**C:** ***n*** = **25 D:** ***n*** = **25 E:** ***n*** = **26**	**C: 36% D: 64% (1 CR) E: 46% (4 CR)**	**C: 7.1 (95% CI, 4.2–8.3) D: 8.4 (95% CI, 4.7–11) E: 5.7 (95% CI, 4.4–14.8)**	**C: 12.9 (95% CI, 8.8-NE) D: 19.3 (95% CI, 14.7–27.4) E: 14.7 (95% CI, 4.4–14.8)**
NCT02031458^b^ (BIRCH) II	PDL-1 positive, IIIB, IV, or recurrent NSCLC	**1L: Atez monotherapy** 2L: Atez monotherapy3L: Atez monotherapy	**1L:** ***n*** = **139** 2L: *n* = 268 3L: *n* = 252	**1L: 22% (95% CI, 15–29)** 2L: 19% (95% CI, 15–25) 3L: 18% (95% CI, 13–23)	1L^**^: 5.4 (95% CI, 3–6.9) 2L^†^: 2.8 (95% CI, 1.5–3.9) 3L^†^: 2.8 (95% CI, 2.7–3.0)	**1L: 23.5 (95% CI, 18.1-NE)** 2L: 15.5 (95% CI, 12.3–19.3) 3L: 13.2 (95% CI, 10.3–17.5)
NCT01846416^b^ (FIR) II	PDL-1 positive, IIIB, IV, or recurrent NSCLC	**1L: Atez monotherapy** 2L: Atez monotherapy 3L: Atez monotherapy	**1L:** ***n*** = **31** 2L: *n* = 71 3L: *n* = 12	1L^***^: 29% (95% CI, 13–45) 2L^†^: 17% (95% CI, 8–26) 3L: 17% (95% CI, 0–38)	N/A	N/A
NCT02525757^a^ II	Non-metastatic, unresectable NSCLC	Group 1: PC + XRTX, *then* atez Group 2: PC + XRTX + atez	N/A^††^	N/A	N/A	N/A
NCT02366143^c^ (IMpower150) III	IV non- squamous NSCLC	Arm A: Atez+PC Arm B: Atez+PC+bev Arm C: PC+bev	A^†††^ B: 356 C: 336	B^***^: 64% (95% CI, 58, 68) C^***^: 48% (95% CI, 43, 54)	B: 8.3 (95% CI, 7.7, 9.8) C: 6.8 (95% CI, 6.0, 7.1)	N/A

*Bolded items represent atezolizumab as first-line therapy*.

a *Currently recruiting participants*.

b *Not yet open for recruiting participants*.

c *Not currently recruiting participants*;

* *Not NSCLC*;

** *IRF-assessed*;

*** *Unconfirmed ORR*;

† *Confirmed ORR*;

†† *Only safety data were reported*;

††† *Data only available for Arms B and C. 1L, cohort 1, atezolizumab as first-line therapy; 2L, cohort 2, atezolizumab as second-line therapy; 3L, cohort 3, atezolizumab as third-line therapy; AEs, adverse events; atez, atezolizumab; bev, bevacizumab; CR, complete remission; DLT, dose-limiting toxicities; INV, investigator; IRF, independent review facility; nab-PC, nab-paclitaxel, carboplatin; NE, not evaluable; NSCLC, non-small cell lung cancer; ORR, objective response rate; PC, paclitaxel, carboplatin; Pem-Cb, pemetrexed-carboplatin; RECIST, Response Evaluation Criteria in Solid Tumors; XRTX, radiation therapy*.

### Studies of atezolizumab as first-line therapy with results

The five studies with results are phase 1b (*n* = 1), phase II (*n* = 3), and phase III (*n* = 1) trials. Endpoints are measured according to the Response Evaluation Criteria in Solid Tumors (RECIST) v1.1 criteria (9).

#### A study of atezolizumab administered in combination with bevacizumab and/or with chemotherapy in participants with locally advanced or metastatic solid tumors (NCT01633970)

In the phase 1b study, atezolizumab was administered in combination with chemotherapy to patients with various tumors (10). Arms A, B, and F included gastric cancer, ovarian cancer, bladder cancer, cervical cancer, and breast cancer. Arms C, D, and E comprised of patients with NSCLC (Table 1). Atezolizumab was administered as maintenance therapy for all arms until disease progression or intolerable toxicity.

Standard chemotherapy doses were used in ≤ 6 cycles and atezolizumab was dosed at 15 mg/kg intravenously (IV) every 3 weeks to determine the maximum tolerated dose (MTD), which was to be used in the dose expansion cohort. Primary outcomes included the MTD, percentage of participants with adverse events (AEs), and percentage of patients with dose-limiting toxicities (DLTs). Secondary outcomes included duration of objective response rate (ORR), progression-free survival (PFS), and the pharmacokinetics of atezolizumab such as clearance, volume of distribution, accumulation ratio, and half-life. The goal enrollment for all arms was 240 patients.

Patient demographics were provided initially for the safety evaluable population but not in the efficacy evaluable population (*n* = 76) (10, 11). Among the patients that were evaluated, the confirmed ORR was the highest in arm D, although the medians and confidence intervals were not provided. Further, Arm D demonstrated the highest median PFS and median overall survival (OS). For adverse effects (AEs), neutropenia and anemia were the most prominent. In arms C, D, and E, 36–42% had neutropenia and 16–31% had anemia. Cases of grade 5 pneumonia, systemic candida, and autoimmune hepatitis in arm E were isolated.

Overall, an interdependent relationship between chemotherapy and immunotherapy was inferred, based on favorable results seen when atezolizumab was given with chemotherapy. Previously, a synergistic effect was seen when pembrolizumab was combined with pemetrexed-carboplatin (12). Despite small enrollment numbers, the study findings led to the initiation of a phase III program—IMpower 130, 131, 132, and 150 (13).

#### BIRCH (NCT02031458)

BIRCH was a phase II, single-arm study that administered atezolizumab in the first- (cohort 1), second- (cohort 2), and third-line (cohort 3) settings in the treatment of advanced NSCLC in patients with a minimum of 5% PDL-1 expression on both tumor cells (TC2/3) and tumor-infiltrating immune cells (IC23), as determined by immunohistochemistry. Patients in cohort 1 had not received previous chemotherapy (in either the locally advanced or metastatic settings). Patients in cohort 2 did receive a platinum-based chemotherapy regimen and progressed, while those in cohort 3 had progressed upon at least two agents. For all three cohorts, atezolizumab was administered at a dose of 1,200 mg IV every 3 weeks until disease progression, unacceptable toxicity, or death. Patients in cohorts 2 and 3 were permitted to continue therapy as long as signs of disease progression and tumor growth were absent, a decline in Eastern Cooperative Oncology Group (ECOG) score was not observed, and clinical benefit was perceived. The primary outcome was the percentage of patients that obtained an independent review facility (IRF)-assessed ORR. Secondary outcome measures included investigator (INV)-assessed ORR as determined by both RECIST v1.1 and modified RECIST, duration of response (DOR), PFS, and OS. [When the unmodified and modified RECIST v1.1 criteria were used in various tumors, the difference in measurements were < 10% and tumor response rates were not statistically significant (14)].

A total of 667 patients were enrolled into the study. Patients with central nervous system (CNS) disease, including metastases, were excluded. Presence of the EGFR mutation or ALK fusion was permitted if previous therapy with a tyrosine-kinase inhibitor was given. The median age was 67 years (range 35–88) in cohort 1, 63 years (range 28–83) in cohort 2, and 64 years (range 38–84) in cohort 3. Men comprised 51% of patients in cohort 1, and 61% in cohorts 2 and 3. The proportion of current or previous tobacco users was similar among the cohorts and ranged from 82 to 84%. The predominant race and histology were white and nonsquamous.

Among the three cohorts, patients in cohort 1 had the highest ORR, median PFS, and median OS (Table 1). When patients with high PDL-1 expression (TC3/IC3) were analyzed, all three measures were the highest in cohort 1 as well. Patients that were previous or current tobacco users and those with nonsquamous histology had the highest response rates. In cohort 1, ORR was achieved by 19% (95% CI, 12–28) in patients that tested negative for the EGFR mutation (*n* = 104) and 23% (95% CI, 5–54) in patients that tested positive (*n* = 13). Similarly, a previous durvalumab trial showed improvement in objective response among patients with advanced NSCLC, ≥25% PDL-1 expression, and with the EGFR mutation or ALK fusion (15).

Further, of the 100 patients analyzed for Kirsten rat sarcoma (KRAS) mutation status in cohort 1, ORR was seen in 27% (95%, 13–46) of patients with the mutation and 16% (95%, 8–27) of patients without the mutation. Cohorts 2 and 3 also showed high ORRs and median PFS for patients regardless of mutation status. The most predominant AEs in all patients were fatigue (19%), diarrhea (11%), and nausea (11%). The total number of AEs and treatment-related AEs were comparable across the cohorts and occurred regardless of PDL-1 expression. AEs that led to the discontinuation of atezolizumab included pneumonitis and pneumonia in 8 patients in cohort 1 (*n* = 139, 6%), 20 patients in cohort 2 (*n* = 268, 8%), and 15 patients in cohort 3 (*n* = 252, 6%). In total, 43 patients (7%) discontinued treatment and 305 deaths (46%) occurred, with 90% of the deaths being attributed to disease progression (16).

#### FIR (NCT01846416)

Like BIRCH, FIR was a phase II, single-arm study that administered atezolizumab as first (cohort 1), second (cohort 2), and third-line (cohort 3) therapy in NSCLC among patients with a minimum PDL-1 expression of 5% (TC2/3 or IC2/3). Cohort 1 constituted patients with no history of chemotherapy in the advanced setting, cohort 2 had patients who progressed during or after a platinum-based regimen, and cohort 3 included patients with progression after an unrestricted number of previous therapies, as well as patients with asymptomatic brain metastases. A total of 138 patients were enrolled in the study and atezolizumab was dosed at 1,200 mg IV every 3 weeks until disease progression. Cohorts 2 and 3 received therapy until investigators did not perceive any clinical benefit. The primary outcome was the INV-assessed ORR according to the modified RECIST v1.1 criteria. Secondary outcomes included INV-assessed ORR, DOR, and percentage of patients with 6-month DOR.

The median age was 66 years, with the range being from 42 to 85. Of all patients, 58% were men. All patients who received at least one dose of atezolizumab were evaluated for efficacy (*n* = 114). The highest ORRs were seen in cohort 1. Of those that responded, the range in DOR was 7–30% in cohort 1 and 11–69% in cohort 2. The median PFS and median OS were not reported, although the 6-month PFS was seen in 39% (95% CI, 22–56) of patients in cohort 1 and 35% (95% CI, 23–46) in cohort 2. The most common treatment-related AEs were fatigue (26%) and nausea (15%), which were considered manageable and tolerable. Grade 3–4 AEs were seen in 15% of patients and included elevated hepatic enzymes, hypertension, and pulmonary embolism. Single cases of Guillian-barre syndrome, diabetes, pneumonitis, and disseminated herpes zoster were reported. Grade 5 AEs occurred in 10 cases, which included cardiac arrest, disseminated intravascular coagulation, and constrictive pericarditis (17).

Based on this data, patients with asymptomatic brain metastases (cohort 3) did respond, though least favorably compared to patients without brain metastases. Previously, in patients with NSCLC and brain metastases, nivolumab showed safety and clinical efficacy, and pembrolizumab demonstrated an acceptable safety profile (18, 19).

#### Atezolizumab with chemoradiation for lung cancer (NCT02525757)

A phase II study was designed to evaluate the response to atezolizumab following, as well as in combination with, chemoradiation (10). The integration of chemoradiation in immunotherapy regimens has been demonstrated in trials with pembrolizumab and nivolumab. This technique is attempted in patients with medically inoperable tumors.

In this trial, Group 1 completed treatment and Group 2 is in the process of patient recruitment (Table 1). Eligible patients had disease that was considered unresectable and non-metastasized with no previous history of chemotherapy. The primary outcome is the time to toxicity, defined as any grade 3 or 4 AE in the first 15 weeks of therapy or any immune-related adverse events (irAEs). The secondary outcome is the PFS at 6 months and 1 year.

Group 1 received atezolizumab 1,200 mg every 3 weeks for up to 14 doses as consolidation therapy after first-line chemoradiation. Standard doses of carboplatin and paclitaxel were used, and radiation was given for 6–7 weeks. Three more patients will be included in this group. Patients in Group 2 will receive atezolizumab in combination with chemoradiation as first-line therapy in approximately 30 patients. The atezolizumab regimen for consolidation and maintenance therapy will mimic that of group 1.

Of the patients that were evaluated for efficacy, 3 experienced irAEs. Grade 3 AEs of arthralgia and dyspnea occurred as isolated cases and disease progression in 2 patients. The AEs were noted to be tolerable and manageable.

#### IMpower150 (NCT02366143)

The phase III study randomized chemotherapy-naïve patients with stage IV non-squamous NSCLC to Arms A, B, or C (Table 1). A total of 356 and 336 patients were enrolled into Arms B and C, respectively, for intention-to-treat wild-type (ITT-WT; EGFR or ALK negative). The primary outcomes were the INV-assessed PFS and OS. Patients with active or untreated CNS metastases or malignancies other than NSCLC within 5 years were excluded. Atezolizumab was dosed at 1,200 mg IV every 3 weeks.

Primary analyses were conducted for Arms B vs. C. The median age was 63 years old and 60% were former smokers in both arms. In arms B and C, 61 and 62% were male. The hazard ratio for PFS in Arm B compared to C was 0.62 (95% CI: 0.52, 0.74; *p* < 0.0001) in the ITT-WT. The median PFS in Arms B and C was 8.3 and 6.8 months, respectively. Patients in Arm B had comparable treatment-related serious AEs to Arm C (25 vs. 19%, respectively) (20).

The design of this trial resembles that of KEYNOTE-021, wherein one of the cohorts of patients with metastatic or unresectable NSCLC received pembrolizumab with chemotherapy and bevacizumab. Unfortunately, all patients in this cohort (*n* = 25) were unable to complete treatment because of disease progression or death. Since patients in IMPOWER150 had less severe disease at baseline, the trial showed the safety and efficacy of adding a checkpoint inhibitor (atezolizumab) to bevacizumab and chemotherapy. Additionally, nivolumab has been safely administered in combination with chemotherapy and bevacizumab as maintenance therapy in patients with advanced NSCLC (21).

### Studies of atezolizumab as first-line therapy without results

For the ten ongoing trials without results, atezolizumab 1,200 mg is administered IV every 3 weeks and the number of cycles is trial-dependent. All studies employ the RECIST v1.1 criteria for the measurement of outcomes. The studies are phase I (*n* = 1), phase II (*n* = 4), and phase III (*n* = 5) trials (Table 2).

**Table 2:** Current trials without results.

**Identifier, phase**	**Disease**	**Intervention**	**Primary outcomes**	**Estimated completion date**
NCT02599454^a^ I	Medically/surgically inoperable I NSCLC	Atez + SBRT	Maximum tolerated dose	November 2019
NCT02994576^a^ (PRINCEPS) II	IB, II, or IIIA (non N2) NSCLC	Atez as neoadjuvant therapy	Rate of patients without major toxicities or morbidities	May 2021
NCT03102242^b^ II	unresectable or inoperable IIIA/B NSCLC	Atez as neoadjuvant therapy, with CRT, and as adjuvant therapy	DCR after 12 weeks induction	Mar 2020
NCT02927301^a^ II	IB, II, or IIIA NSCLC	Atez as neoadjuvant and adjuvant therapy	Major pathologic response based on surgical resection^*^	July 2023
NCT02848651^a^ (B-F1RST) II	IIIB-IVB NSCLC	Atez monotherapy	INV-assessed ORR per modified RECIST v1.1; PFS per RECIST v1.1 by circulating blood-based tumor biomarkers	June 2020
NCT02409342^a^ (IMpower110) III	PDL-1 positive, IV non-squamous or squamous NSCLC	Atez vs. chemotherapy	OS	August 2020
NCT02409355^c^ IMpower111) III	PDL-1 positive, IV squamous NSCLC	Atezo vs. chemotherapy	INV-assessed PFS per RECIST v1.1	September 2017
NCT02367781^c^ (IMpower130) III	IV non-squamous NSCLC	(Atez + chemotherapy) vs. chemotherapy	INV-assessed PFS per RECIST v1.1 (ITT and PDL-1-selected population); OS (ITT and PDL-1-selected population)	October 2018
NCT02367794^c^ (IMpower131) III	IV squamous NSCLC	(Atez + chemotherapy) vs. chemotherapy	INV-assessed PFS per RECIST v1.1 (ITT and TGE population); OS (ITT population)	February 2023
NCT02657434^a^ (IMpower132) III	IV non-squamous NSCLC	(Atez + chemotherapy) vs. chemotherapy	INV-assessed PFS per RECIST v1.1; OS	November 2019

a *Currently recruiting participants*.

b *Not yet open for recruiting participants*.

c *Not currently recruiting participants*.

* *Hellman et al., 2014. Atez, atezolizumab; AEs, adverse events; CRT, chemoradiotherapy; DCR, disease control rate; DLT, dose-limiting toxicities; IC1/2/3, tumor-infiltrating immune cells with minimum 1% PDL-1 expression; IC2/3, tumor-infiltrating immune cells with minimum 5% PDL-1 expression; INV, investigator; IRF, independent review facility; ITT, intent-to-treat; NSCLC, non-small cell lung cancer; ORR, objective response rate; OS, overall survival; PFS, progression-free survival; RECIST, Response Evaluation Criteria in Solid Tumors; SBRT, stereotactic ablative radiotherapy; TC1/2/3, tumor cells with minimum 1% PDL-1 expression; TC2/3, tumor cells with minimum 5% PDL-1 expression; TGE, tumor gene expression*.

#### Atezolizumab and stereotactic body radiation therapy in treating patients with non-small cell lung cancer (NCT02599454)

A single-group phase I study is administering atezolizumab in combination with stereotactic body radiation therapy (SBRT) in stage I NSCLC to 33 patients who are not medical or surgical candidates. Eligible patients must have a tumor size ≤ 5 cm. During the dose escalation phase, patients receive atezolizumab for 6 courses. After course 3, SBRT is administered in 4–5 fractions. During the expansion phase, atezolizumab and SBRT are given in a similar manner but atezolizumab is continued until signs of toxicity or disease progression. The primary outcome is the maximum tolerated atezolizumab dose that can be given concomitantly with SBRT.

Previously, a case report showed that patients with brain metastases responded better to nivolumab following radiotherapy, compared to before (22). Further, in patients with metastatic NSCLC, pembrolizumab in combination with SBRT did not show an increase in toxicity (23). Based on previous experience with other immune checkpoint inhibitors, the results of this trial appear promising.

#### Atezolizumab as induction therapy in non-small cell lung cancer (PRINCEPS) (NCT02994576)

In the single-arm, phase II study, atezolizumab is being administered as neoadjuvant therapy in approximately 60 patients with stage 1B to IIIA (non N2) NSCLC. Eligible patients must have disease that is considered resectable. Patients receive atezolizumab as a one-time IV infusion. The primary outcome is the rate of patients that do not experience major toxicities or morbidities until 1 month following surgical resection.

#### Atezolizumab immunotherapy in patients with advanced NSCLC (NCT03102242)

Patients with newly diagnosed and unresectable stage IIIA/B NSCLC are receiving atezolizumab in combination with chemoradiotherapy in a single-arm phase II pilot trial. An estimated number of 63 patients are enrolled. Atezolizumab is being administered in 4 cycles and patients will be re-staged following the second and fourth cycles. If any signs of disease progression occur after the second cycle, atezolizumab will be discontinued and chemoradiotherapy initiated. The primary outcome is the rate of disease control after 12 weeks of induction therapy. Eligible patients will receive up to 1 year of adjuvant therapy with atezolizumab. Previously, most patients with stage III NSCLC safely received consolidation with pembrolizumab following chemoradiation with low risk for serious pneumonitis (24).

#### A study of atezolizumab as neoadjuvant and adjuvant therapy in stage IB, II, or IIIA resectable and untreated non-small cell lung cancer (NSCLC) (NCT02927301)

Approximately 180 patients with stage 1B to IIIA resectable NSCLC are being given atezolizumab as neoadjuvant and adjuvant therapy in a single-arm phase II trial (25). Eligible patients must have adequate end-organ and hematologic function. In the neoadjuvant setting, atezolizumab is being given for 2 cycles and eligible patients continue to receive adjuvant atezolizumab for up to a year. The primary outcome is the major pathologic response following surgery, i.e. an amount of viable tumor that is <10%. Note that following neoadjuvant chemotherapy, patients with a viable tumor of ≤ 10% had longer OS and disease-free survival (DFS) compared to those with ≥10% viable tumor (26). In a recent small study, neoadjuvant nivolumab resulted in ≤ 10% viable tumor in 45% of patients with resectable stage I-III NSCLC (*n* = 20) (27). It is the expectation that atezolizumab would yield similar survival benefits.

#### A study of atezolizumab as first-line monotherapy for advanced or metastatic non-small cell lung cancer (NSCLC): clinical evaluation of novel blood-based diagnostics [B-F1RST] (NCT02848651)

Novel biomarker assays are being used instead of standard tumor biopsies to measure PDL-1 expression in a single-arm, phase II study (28). However, using PDL-1 as a biomarker is controversial, as studies employ dissimilar criteria for detecting PDL-1 positivity, as well as different methods for sample collection and staining (29).

Approximately 150 patients with stage IIIB to IVB NSCLC regardless of PDL-1 expression are being administered atezolizumab as monotherapy until disease progression or intolerable toxicity. Adequate end-organ function and hematologic function are required. Patients with the EGFR mutation or ALK fusion and active CNS metastases are excluded. Co-primary outcomes include INV-assessed ORR and PFS based on retrospective analysis of tumor biomarkers.

#### IMpower110, 111, 130, 131, and 132 (NCT02409342, NCT02409355, NCT02367781, NCT02367794, NCT02657434)

The IMpower trials are double-arm, open-label, phase III studies that compare standard chemotherapy with atezolizumab as monotherapy (IMpower110 and 111) or in combination with standard chemotherapy (IMpower130, 131, and 132). Eligible patients for all IMpower trials have stage IV NSCLC, an ECOG score of 0–1, and adequate end organ function. Patients receive atezolizumab until no clinical improvement or disease progression, along with standard chemotherapy doses. The primary endpoints are clinically relevant outcomes such as PFS and OS.

All patients in IMpower110 and 111 have tumors that are positive for PDL-1 expression. IMpower110 randomizes patients with non-squamous histology in a one-to-one manner to either atezolizumab or platinum-based chemotherapy containing pemetrexed. Those with squamous histology are randomized one-to-one to atezolizumab or platinum-based chemotherapy with gemcitabine. IMpower111 is a smaller phase III trial that is administering atezolizumab to patients exclusively with squamous cell NSCLC. The drug and regimen are identical to IMpower110 for this histology.

IMpower130, 131, and 132 are large phase III trials that are comparing atezolizumab in combination with chemotherapy to standard chemotherapy. IMpower130 assigns patients two-to-one to either the experimental arm (platinum-based chemotherapy in combination with atezolizumab) or the comparator arm (standard platinum-based chemotherapy) (30). IMpower131 uses the same regimen as IMpower130, with an additional arm of nab-paclitaxel in the experimental group (13). For maintenance therapy, atezolizumab is continued in the experimental arm and best supportive care in the comparator arm. The IMpower studies measure clinically relevant outcomes such as PFS and OS as the primary endpoint and will ascertain the role of atezolizumab in NSCLC.

## Discussion

Recently, the single-arm BIRCH trial showed high rates of OS, PFS, and ORR for atezolizumab in chemotherapy-naïve patients with at least 5% PDL-1 expression (16). In addition, the smaller single-arm FIR trial also showed high ORR in patients with a minimum of 5% PDL-1 expression when given atezolizumab as first-line therapy, although the long-term clinical benefits such as PFS and OS are not known yet (17). Whether atezolizumab will gain approval for first-line therapy in a similar fashion to pembrolizumab will be determined by trials such as IMpower110 and 111, which will randomize chemotherapy-naive patients to either atezolizumab or standard platinum-based therapy in PDL-1 positive patients with NSCLC (31).

When atezolizumab was given as first-line therapy in combination with chemotherapy, impressive response rates were seen (10). The highest ORR of 64% was seen in patients that received atezolizumab with carboplatin and pemetrexed. In a previous pembrolizumab trial, the ORR was 29% (95% CI, 18–41) in patients that received carboplatin and pemetrexed, and 55% (95% CI, 42–68) in patients that received this regimen in addition to pembrolizumab (12). However, it is evident that direct comparisons cannot be made between the two trials because of the use of different study methodologies and unidentical patient populations. Most importantly, the phase 1b atezolizumab study did not report interquartile ranges for the results.

The mechanism for the possible synergy between PDL-1 inhibitors and chemotherapy is not clear. In NSCLC, chemotherapy decreased the level of PDL-1 expression on tumor cells, and an increased level of PDL-1 expression was shown to be an independent risk factor for decreased overall survival (32, 33). However, discrepancies existed in other cancers especially breast cancer (34). Chemotherapeutic agents such as paclitaxel increased the expression of PDL-1, whereas doxorubicin decreased the expression of PDL-1 (34, 35).

The administration of atezolizumab after chemoradiation was shown to be safe in patients with non-metastatic, unresectable NSCLC (36). A previous study showed that radiation could induce PDL-1 expression on cells, thereby rendering PDL-1 inhibitors more effective (37). Thus, chemoradiation may increase the potency of atezolizumab. In the trials to come, atezolizumab will be administered as neoadjuvant therapy in NSCLC as early as stage IB, and as such, the patient population that could benefit from atezolizumab may increase. Further, new blood-based biomarker assays will be used to determine PDL-1 expression and predict treatment response. If the assays denote accuracy and reliability, tumor biopsies may not be necessary for patients.

Overall, atezolizumab has shown a clinical benefit in the first-line setting of NSCLC regardless of histology, PDL-1 expression, and EGFR or KRAS mutation status. High rates of response and tolerability have been demonstrated. Further evidence from ongoing and future trials will precisely determine the role of atezolizumab in the treatment of NSCLC.

## Author contributions

RR and KW contributed to the conception and design of the study, organized the database, and wrote the manuscript. Both authors contributed to manuscript revision, read and approved the submitted version.

### Conflict of interest statement

The authors declare that the research was conducted in the absence of any commercial or financial relationships that could be construed as a potential conflict of interest.
