# Supplementary material for: Atezolizumab for the First-Line Treatment of Non-small Cell Lung Cancer (NSCLC): Current Status and Future Prospects
Source: Front Oncol. 2018 Jul 24;8:277. doi: 10.3389/fonc.2018.00277 (PMC6066722; doi:10.3389/fonc.2018.00277)
Supplement: Supplementary file 1 [file Data_Sheet_1.docx]

**Appendix 1:** Pubmed search strategy (search performed on 5/17/17):

| 1 | "nonsmall cell lung cancer"[mesh:noexp] |
| --- | --- |
| 2 | "Carcinoma, Squamous Cell"[mesh:noexp] |
| 3 | "Carcinoma, Large Cell"[mesh:noexp] |
| 4 | "Carcinoma, Adenosquamous"[mesh:noexp] |
| 5 | "nonsmall cell lung cancer"[tw] |
| 6 | "NSCLC" [tw] |
| 7 | "squamous cell cancer"[tw] |
| 8 | "large cell carcinoma"[tw] |
| 9 | "adenosquamous carcinoma"[tw] |
| 10 | 1 OR 2 OR 3 OR 4 OR 5 OR 6 OR 7 OR 8 OR 9 |
| 11 | "atezolizumab"[nm] |
| 12 | "atezolizumab"[tw] |
| 13 | "Tecentriq"[tw] |
| 14 | "MPDL3280A"[tw] |
| 15 | 11 OR 12 OR 13 OR 14 |
| 16 | 10 AND 15 |
